# A Case-Crossover Study of Heat Exposure and Injury Risk in Outdoor Agricultural Workers

**DOI:** 10.1371/journal.pone.0164498

**Published:** 2016-10-07

**Authors:** June T. Spector, David K. Bonauto, Lianne Sheppard, Tania Busch-Isaksen, Miriam Calkins, Darrin Adams, Max Lieblich, Richard A. Fenske

**Affiliations:** 1 Department of Environmental and Occupational Health Sciences, University of Washington, Seattle, Washington, United States of America; 2 Department of Medicine, University of Washington, Seattle, Washington, United States of America; 3 Safety and Health Assessment and Research for Prevention (SHARP) Program, Washington State Department of Labor and Industries, Olympia, Washington, United States of America; 4 Department of Biostatistics, University of Washington, Seattle, Washington, United States of America; 5 Department of Mathematics, University of Washington, Seattle, Washington, United States of America; University at Buffalo - The State University of New York, UNITED STATES

## Abstract

**Background:**

Recent research suggests that heat exposure may increase the risk of traumatic injuries. Published heat-related epidemiological studies have relied upon exposure data from individual weather stations.

**Objective:**

To evaluate the association between heat exposure and traumatic injuries in outdoor agricultural workers exposed to ambient heat and internal heat generated by physical activity using modeled ambient exposure data.

**Methods:**

A case-crossover study using time-stratified referent selection among 12,213 outdoor agricultural workers with new Washington State Fund workers’ compensation traumatic injury claims between 2000 and 2012 was conducted. Maximum daily Humidex exposures, derived from modeled meteorological data, were assigned to latitudes and longitudes of injury locations on injury and referent dates. Conditional logistic regression was used to estimate odds ratios of injury for *a priori* daily maximum Humidex categories.

**Results:**

The mean of within-stratum (injury day and corresponding referent days) standard deviations of daily maximum Humidex was 4.8. The traumatic injury odds ratio was 1.14 (95% confidence interval 1.06, 1.22), 1.15 (95% confidence interval 1.06, 1.25), and 1.10 (95% confidence interval 1.01, 1.20) for daily maximum Humidex of 25–29, 30–33, and ≥34, respectively, compared to < 25, adjusted for self-reported duration of employment. Stronger associations were observed during cherry harvest duties in the June and July time period, compared to all duties over the entire study period.

**Conclusions:**

Agricultural workers laboring in warm conditions are at risk for heat-related traumatic injuries. Combined heat-related illness and injury prevention efforts should be considered in high-risk populations exposed to warm ambient conditions in the setting of physical exertion.

## Introduction

Adverse health effects from heat exposure are of public health concern, particularly for populations vulnerable to heat, including the elderly, workers and athletes engaging in physically demanding activities, and others with social and physiologic vulnerabilities [1]. One direct and well-documented adverse health consequence of heat exposure is heat-related illness, which ranges from heat rash to more severe heat exhaustion and heat stroke. Heat stroke, which can be fatal, is characterized as classical or exertional, in which internal metabolic heat generated by physical work additionally contributes to overall heat stress [2].

The burden of heat health effects has been investigated in a variety of populations, including the general population and occupational populations. In the general population, heat waves, which are projected to increase in frequency and severity with climate change [3], have been reported to be associated with increased all-cause mortality, emergency medical services calls, emergency department visits, and hospital admissions for multiple outcomes, including heat-related illness and dehydration, renal disease, diabetes, and obstructive lung disease [1,4–6]. In occupational populations, data from the United States (US) Bureau of Labor Statistics (BLS) indicate that 359 heat-related deaths occurred between 2000 and 2010, with the highest rate in the agricultural industry (mean heat-related death rate of 3.1 per million workers per year; rate ratio 35.2 [95% confidence interval 26.3–47.0], compared to all industries) and among Hispanics [7]. Non-fatal occupational heat-related illness has also been characterized using such sources as workers’ compensation data [8].

Heat exposure in outdoor working populations may increase the risk of traumatic injuries. Traumatic injuries are of particular interest in industries such as agriculture and construction, as these industries are among the US industries with the highest rates of fatal injuries [9]. A descriptive study of Washington State Fund workers’ compensation claims for injuries occurring in orchards from 1996 to 2001 reported that ladder-related claims, including claims for falls from ladders during physically demanding tree fruit harvest activities, accounted for approximately half of claims involving more than medical treatment and were the most expensive (mean annual cost of $3.6 million), compared to claims accepted for other causes [10].

Results from human studies in laboratory settings provide biological plausibility for an association between heat exposure and traumatic injuries, in the context of physical activity. Exercise-related mild dehydration (mean percent body mass loss 1.6%) without hyperthermia has been reported to be associated with adverse changes in vigilance in men [11]. Mild dehydration has been reported to be associated with reduced Profile of Mood States concentration scores in women [12]. Post-exercise balance impairments are hypothesized to be affected by such factors as fatigue, dehydration, inner ear changes, and hyperthermia [13,14]. In addition, sweating may affect grip [15], for example when climbing ladders, and other manual tasks.

Several studies have reported an association between heat exposure and injuries. Morabito et al reported an association between warm weather (average daytime heat index 25–28°C) and increased hospital admissions for work-related accidents from June to September, 1998 and 2003, in Central Italy using meteorological data from one weather station [16]. A study in Quebec, Canada found an incidence rate ratio of daily workers’ compensation claims for acute injuries per 1°C increase in maximum daily temperature from May to September, 2003 to 2010, of 1.002 (95% CI 1.002 to 1.003) using data from one weather station per health region [17]. The incidence rate ratio of injury claims in agriculture per 1°C in maximum daily temperature was 1.005 (95% confidence interval 0.993 to 1.016). A similar study in Adelaide, Australia also found a 0.2% increase in daily injury claims with an increase of 1°C daily maximum temperature for temperatures between 14.2°C and 37.7°C (incidence rate ratio 1.002, 95% confidence interval 1.001 to 1.004; agriculture, forestry, fishing incidence rate ratio 1.007, 95% confidence interval 1.001 to 1.013) using data from one weather station [18]. These studies may be subject to exposure misclassification, as they relied upon meteorological data from relatively few weather stations.

The primary aim of this study was to more closely examine the relationship between heat exposure and injuries by assessing the association between heat exposure and workers’ compensation traumatic injuries in Washington State outdoor agricultural workers using modeled ambient exposure data. The secondary aim was to assess this relationship specifically for tree fruit harvest, which is associated with a large number of injuries, typically involves tasks that do not vary substantially over the harvest period, is physically intense (rate of energy expenditure of approximately 300 Watts, as estimated using standard methods [19]), and occurs primarily during the summer months. We hypothesized that there is a positive association of increased Washington agriculture workers’ compensation traumatic injuries with increased daily maximum apparent temperature, particularly during tree fruit harvest in warm weather, but that at very high apparent temperatures, risk declines, potentially as a result of health protective work practices in very hot weather in this industry.

## Materials and Methods

A case-crossover study using time-stratified referent selection was conducted to assess the relationship between maximum daily Humidex, an apparent temperature calculated from air temperature and dew point [20], and Washington State Fund workers’ compensation traumatic injury claims between 2000 and 2012. A case-crossover design was selected in part because it does not require denominator data. In Washington, the agricultural worker population includes seasonal and migrant workers, such as workers who have obtained H-2A visas to travel for example from Mexico to the US, to perform temporary agricultural work [21,22]. The total number of Washington agricultural workers, which includes workers who may be undocumented but are still eligible to file workers’ compensation claims for work-related injuries, is difficult to confidently enumerate with high temporal resolution. A case-crossover study design, in which exposures at the time of injury are compared to exposures at control times (cases serve as their own “controls”), has advantages of avoiding control selection bias and addressing time-invariant confounders in the design.

### Adult outdoor agriculture traumatic injuries

Injury workers’ compensation claims were identified from the Washington State Department of Labor and Industries (L&I) industrial insurance system claims databases using the methods described in the S1 Supplemental Material. A total of 780,499 traumatic injury workers’ compensation claims were identified from 1,095,533 injury claims with injury dates between January 1, 2000 and December 31, 2012 that occurred either at the worksite or on the employer’s premises (see S1 Supplemental Material, p. 2–3 and S1 Fig).

Available workers’ compensation claims data included fields for accident addresses, employer business location addresses, and addresses of the first healthcare provider visited by the claimant. Latitude and longitude were assigned to all available addresses for each claim using Geocoder::US 2.0 [23] and US Census TIGER/LINE^®^ 2014 reference data [24]. An *a priori* scheme was used to assign a single location per claim as the injury location to which exposures were assigned (see S1 Supplemental Material, p. 3).

New adult outdoor agriculture traumatic injury cases were defined using the scheme described in S2 Fig and in the S1 Supplemental Material, p. 3–5. Claims in Western Washington were excluded from the primary analysis for two reasons: 1) the majority of Washington crop agriculture occurs east of the Cascades Mountains, rather than in Western Washington; and 2) the Cascade Mountains divide Washington into two main climatic areas [25]. Compared to Western Washington, summers are warmer and drier east of the Cascades. A total of 12,213 new Central/Eastern Washington adult traumatic outdoor agriculture injury cases were available for analysis.

### Referent selection

Exposure levels on injury days were compared with exposure levels on referent days, defined as the same day of the week during the month of injury, at the same location (time-stratified referent selection [26]). Potential referent days within the month of injury were excluded if the claimant was not working for the employer of injury during the quarter of and the quarter before the injury, based on Washington Employment Security Department data (see S1 Supplemental Material, p. 2), and the day fell on a date that preceded the date a claimant reported starting work for the employer of injury (1.3% of days). Although some claimants were expected to be off work for medical or other reasons for more than a week after injury, referent days after injury were not excluded from analyses for the following reasons. In the extreme case of fatality outcomes (the probability of a fatal event occurring twice is zero), bias in effect estimates associated with including referent days after the event is proportional to time trends in exposure, which are minimized by using time stratified referent selection [26], and the unconditional probability of the outcome [27]. The probability of a traumatic injury at a given time is expected to be low. Time stratified referent selection avoids overlap bias, which can be larger than bias related to the inclusion of referent days after the event [26]. The mean number of referent days per injury day was 3.2.

### Exposure and spatiotemporal join

Maximum daily Humidex, derived from University of Washington Climate Impacts Group gridded meteorological data, based on parameter-elevation relationships of independent slopes models (PRISM) and National Oceanic and Atmospheric Administration global historical climate network daily data and geographic features, with exposures assigned to the centroid of each grid rectangle, was used [28]. The model’s spatial resolution is 1/16th degree latitude by longitude, which corresponds to approximately 7.0 by 4.5 km in the study area. Exposure and outcome data for the injury and referent dates at the corresponding injury location were joined using a Euclidean nearest neighbor approach with ArcGIS (version 10.2.0.3348) (Esri, Redlands, CA). One and two day exposure lags were not considered, as previous studies have not identified an increased risk of injury associated with these lags [17,18]. Exposure levels were categorized *a priori* (<25, 25–29, 30–33, ≥34) based on the Occupational Health Clinics for Ontario Workers Humidex Based Heat Response Plan [29], which is adapted from the American Conference of Governmental Industrial Hygienist (ACGIH) Heat Stress Threshold Limit Value (TLV)^®^ [19].

### Statistical analysis

Odds ratios of injury for daily maximum Humidex categories, compared to the reference category of <25, were estimated in primary analyses using conditional logistic regression, maximizing the exact conditional likelihood, with the *clogit* function in the *survival* R package (version 2.38–3). Models were adjusted for duration of employment, which was calculated for injury days and for referent days by subtracting or adding the number of days between the referent date and injury date to the self-reported duration of employment at the time of injury for referent dates before and after the injury date, respectively.

In secondary analyses, continuous and dichotomous (≥25 versus <25) maximum daily Humidex exposures were explored and analyses were stratified by cherry and apple harvest duties. Washington is the top producer of apples (harvested primarily in August, September, and October) and cherries (harvested primarily in June and July) in the US, and tree fruit harvest most commonly requires the use of ladders [30]. Given a hypothesized mechanism of association between heat exposure and ladder injuries involving dehydration and reduced vigilance, analyses were stratified by cherry harvest duties (96% of cherry harvest injuries occurred during the months of June and July) and apple harvest duties (94% of apple harvest injuries occurred during August, September, and October). Job duties were characterized from the employer of injury job title/duties free text field filled out by the claimant on the report of accident claim form (see S1 Supplemental Material, p. 2). Relatively fewer injuries that occurred during the study period were associated with apple tree pruning (n = 46 injuries), apple tree thinning (n = 102 injuries), or pear or peach harvest (n = 138 injuries) than with apple harvest (n = 1,043 injuries) or cherry harvest (n = 571 injuries). We selected cherry and apple harvest duties for secondary analyses because they had the highest frequencies of tree fruit harvest injuries. A secondary analysis was also performed for the May to September period rather than the entire calendar year, as the May to September time period is thought to be the highest risk time period for occupational heat health effects in Washington State [8]. Workplace safety standards intended to address outdoor heat in agriculture are currently in effect from May to September in Washington State (Washington Administrative Code 296-307-097) [31].

Several sensitivity analyses were performed, excluding from primary analyses injuries (and corresponding referent days): that occurred on weekends; that occurred on US public holidays (using the *holiday* function in the timeDate package in R 3.2.3 [R Foundation, Vienna, Austria] [32]); with locations based only on zip code or city (see S1 Supplemental Material, p. 4); with injury locations based only on first healthcare address (see S1 Supplemental Material, p. 3); with geocoding accuracy scores less than 0.80 (see S1 Supplemental Material, p. 3–4); with injury times that were not between 5:30 am and 4:30 pm; with injury times that were not between 5:30 am and 12:30 pm; with more than seven days of time-loss (lost work time due to work-related injury or illness after a three day waiting period); with more than one day between the injury date and the first visit to a healthcare provider; and that resulted in death. Analyses were also repeated with maximum daily dry air temperature instead of Humidex

All analyses were performed using R 3.2.3 (R Foundation, Vienna, Austria) [32]. The Washington State Institutional Review Board reviewed the study protocol and determined the study to be exempt.

## Results

### Traumatic injury claim characteristics

Characteristics of the 12,213 injury claims are shown in Table 1. There were between 664 (in 2003) and 1,275 (in 2000) traumatic injuries per year during the study period. Only 19 injury claims (0.2%) met the criteria for heat-related illness, as previously defined [33]. Frequencies of traumatic injuries by harvest duty types and peak harvest month are shown in S1 Table. Characteristics of cherry harvest duty injuries during June and July (n = 546) were similar to characteristics for the entire study population, except the median (interquartile range) self-reported duration of employment at the employer of injury was shorter for cherry harvest duties (7 [2, 30] versus 61 [7, 760] days for the entire study population). A smaller percentage of cherry harvest duty traumatic injury claims occurred in males (52% versus 78%), and a larger percentage involved multiple body parts (29% versus 14%) and occurred as a consequence of a fall (83% versus 48%). A larger percentage of cherry harvest duty claims involved three or more days of missed work (36% versus 26%).

**Table 1:** Injury claim characteristics (N = 12,213).

Characteristic	n (%) or median (interquartile range)
Age (years)	
18–34	6,929 (57%)
35–44	2,762 (23%)
45–54	1,638 (13%)
55–64	681 (6%)
65+	203 (2%)
Male gender	9,468 (78%)
Body mass index (kg/m^2^)^a^	27 (24, 30)
Self-reported duration of employment at employer of injury (days)	61 (7, 760)
Industry^b^	
Apple orchards	3,816 (31%)
Other non-citrus fruit farming	2,457 (20%)
All other miscellaneous crop farming	1,110 (9%)
Injury day of the week	
Monday	2,211 (18%)
Tuesday	2,060 (17%)
Wednesday	2,004 (16%)
Thursday	1,966 (16%)
Friday	2,017 (17%)
Saturday	1,286 (11%)
Sunday	669 (5%)
Injury month	
January	473 (4%)
February	563 (5%)
March	678 (6%)
April	671 (5%)
May	636 (5%)
June	1,436 (12%)
July	1,527 (13%)
August	1,411 (12%)
September	2,320 (19%)
October	1,616 (13%)
November	507 (4%)
December	375 (3%)
Body part^b^	
Upper extremity	4,717 (39%)
Lower extremity	2,709 (22%)
Trunk	2,628 (22%)
Injury source/cause^b^	
Structures & surfaces	4,480 (37%)
Person, plants, animals, & minerals (e.g. bodily motion of injured worker)	2,223 (18%)
Tools, instruments, & equipment	1,609 (13%)
Injury nature^b^	
Surface wounds & bruises	3,837 (31%)
Muscles, tendons, ligaments, & joints	3,084 (25%)
Open wounds	2,284 (19%)
Injury event/exposure^b^	
Falls	5,893 (48%)
Bodily reaction & exertion	3,947 (32%)
Other events/exposures	831 (7%)
Claim status	
Compensable^c^	3,226 (26%)
Time-loss, compensable^c^ claims (days)	26 (3,136)
Days between injury and first healthcare visit^d^	
0	7,278 (60%)
1	1,790 (15%)
2	652 (5%)
Location upon which injury location assignment based	
Accident location	8,557 (70%)
Business location (business location and location of first healthcare provider in same county)	1,931 (16%)
First healthcare provider location	1,725 (14%)
Granularity of injury locations	
Full address	6,985 (57%)
Street	745 (6%)
Intersection	2 (0%)
City	891 (7%)
Zip code	3,590 (29%)

^a^ 5,613 observations missing

^b^ Top three by prevalence

^c^ More than medical treatment only (e.g. time-loss compensation)

^d^ 357 observations missing

### Heat exposure characteristics

The mean (standard deviation) and median (interquartile range) daily maximum Humidex on injury and referent days at injury locations are shown in Table 2. The mean (range) maximum daily dry air temperature during the May to September time period was 27.9°C (8.0°C, 41.9°C). The mean of within-stratum (injury day and corresponding referent days) standard deviations of maximum daily Humidex at injury locations was 4.8. Table 2 additionally shows the number of strata (injury and corresponding referent days) that contained the different categories of Humidex exposures and the number of injury and referent days within each Humidex category. A plot of locations of injuries by exposure level did not suggest an obvious spatial trend in exposures (see S3 Fig). Approximately 7,000 injuries (60%) occurred between 5:30 am and 12:30 pm on injury days, with 54% of these morning injuries occurring on days when the maximum daily Humidex was less than 25, 19% on days when the maximum daily Humidex was between 25 and 29, 12% on days when the maximum daily Humidex was between 30 and 33, and 14% occurring on days when the maximum daily Humidex was 34 or greater.

**Table 2:** Maximum daily Humidex characteristics for adult outdoor agriculture traumatic injury days and referent days.

All	Mean (SD) maximum daily Humidex	Median (IQR)maximum daily Humidex	Number of strata containing Humidex category^a^ (number of days within each Humidex category: injury; referent)^b^
Injury days (n = 12,213)	21.4 (11.1)	22.7 (14.0, 29.7)	—
Referent days (n = 39,588)	21.0 (11.4)	22.1 (13.4, 29.5)	—
Mean of within strata SDs = 4.8	—	—	—
Humidex categories	—	—	
<25			9,818 (7,077; 23,614)
25–29			5,743 (2,229; 6,798)
30–33			4,064 (1,399; 4,222)
≥34			3,497 (1,508; 4,954)
**May-Sept only**			
Injury days (n = 7,330)	28.3 (6.6)	28.2 (23.6, 33.0)	—
Referent days (n = 23,553)	28.2 (6.8)	28.0 (23.3, 33.0)	—
Mean of within strata SDs = 5.0	—	—	—
Humidex categories			
<25			4,938 (2,346; 7,929)
25–29			5,333 (2,088; 6,479)
30–33			4,023 (1,388; 4,192)
≥34			3,496 (1,508; 4,953)
**Cherry harvest duties, Jun-Jul**			
Injury days (n = 546)	30.8 (5.8)	30.4 (26.4, 34.8)	—
Referent days (n = 1,634)	29.8 (6.4)	29.4 (25.1, 34.5)	—
Mean of within strata SDs = 5.2	—	—	—
Humidex categories			
<25			307 (94; 397)
25–29			390 (164; 488)
30–33			321 (130; 310)
≥34			332 (158; 439)
**Apple harvest duties, Aug-Oct**			
Injury days (n = 981)	21.9 (7.0)	21.8 (16.7, 27.1)	—
Referent days (n = 3,052)	21.3 (7.8)	21.0 (15.9, 26.8)	—
Mean of within strata SDs = 4.7	—	—	—
<25			884 (653; 2,064)
25–29			488 (201; 588)
30–33			264 (88; 244)
≥34			117 (39; 156)

IQR interquartile range; SD standard deviation

^a^ Injury day and/or at least one referent day within the stratum falls within Humidex category

^b^ Number of injury days and referent days within each Humidex category do not sum to the number of strata containing each Humidex category; each stratum contains more than one referent day

### Association of heat exposure with traumatic injuries

**Fig 1:**
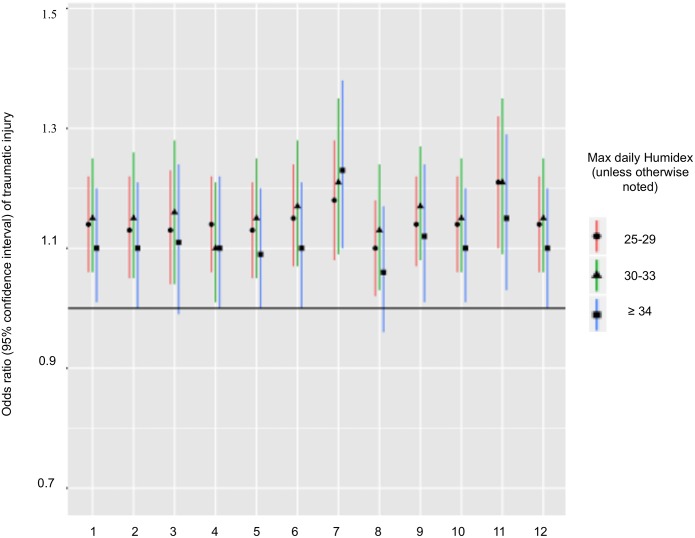
Sensitivity analyses. 1 primary analysis (N = 51,801); 2 sensitivity analysis excluding from primary analyses injuries (and corresponding referent days) that occurred on weekends (included n = 43,531); 3 sensitivity analysis excluding from primary analyses injuries (and corresponding referent days) with locations based only on zip code or city (included n = 32,810); 4 sensitivity analysis excluding from primary analyses injuries (and corresponding referent days) with injury locations based only on first healthcare address (included n = 44,498); 5 sensitivity analysis excluding from primary analyses injuries (and corresponding referent days) with geocoding accuracy scores less than 0.80 (included n = 47,588); 6 sensitivity analysis excluding from primary analyses injuries (and corresponding referent days) with injury times that were not between 5:30 am and 4:30 pm (included n = 43,808); 7 sensitivity analysis excluding from primary analyses injuries (and corresponding referent days) with injury times that were not between 5:30 am and 12:30 pm (included n = 30,870); 8 sensitivity analysis excluding from primary analyses injuries (and corresponding referent days) with more than seven days of time-loss (included n = 42,885); 9 sensitivity analysis with maximum daily dry air temperature instead of Humidex (N = 51,801); 10 sensitivity analysis excluding from primary analyses injuries (and corresponding referent days) that resulted in death (included n = 51,776); 11 sensitivity analysis excluding from primary analyses injuries (and corresponding referent days) with more than one day between the injury date and the first visit to a healthcare provider (included n = 30,758); 12 sensitivity analysis excluding from primary analyses injuries (and corresponding referent days) with injuries that occurred on public holidays (n = 49,713).

Table 3 shows odds ratios (OR) and 95% confidence intervals (CI) of workers’ compensation traumatic injuries. Results of the primary analysis indicated a traumatic injury OR of 1.14 (95% CI 1.06, 1.22) for daily maximum Humidex of 25–29, 1.15 (95% CI 1.06, 1.25) for daily maximum Humidex of 30–33, and 1.10 (95% CI 1.01, 1.20) for daily maximum Humidex of 34 or greater, compared to daily maximum Humidex < 25, adjusted for self-reported duration of employment at the job of injury. A greater self-reported duration of employment was associated with a decreased risk of traumatic injury, after adjustment for heat exposure (OR 0.994; 95% confidence interval 0.992, 0.996). The mean of within-stratum (injury day and corresponding referent days) standard deviations of self-reported duration of employment at the employer of injury was 9.6 days. Effect estimates were generally higher during cherry harvest in the June and July time period (Table 3). Results of other secondary analyses are shown in Table 3, and results of sensitivity analyses are shown in Fig 1. Results of sensitivity analyses using maximum dry air temperature instead of Humidex were similar to results for the main analysis.

**Table 3:** Odds ratios (ORs) and 95% confidence intervals (CIs) of workers’ compensation traumatic injuries.

All (N = 51,801)^a^	Unadjusted OR (95% CI)	Adjusted OR (95% CI)^b^
H_max_ (ref: <25) (n = 30,691)	1.00	1.00
25–29 (n = 9,027)	1.14 (1.06, 1.22)	1.14 (1.06, 1.22)
30–33 (n = 5,621)	1.15 (1.05, 1.24)	1.15 (1.06, 1.25)
34 or greater (n = 6,462)	1.10 (1.01, 1.20)	1.10 (1.01, 1.20)
H_max_ (ref: <25) (n = 30,691)	1.00	1.00
25 or greater (n = 21,110)	1.13 (1.07, 1.21)	1.13 (1.07, 1.20)
H_max_	1.01 (1.01, 1.02)	1.01 (1.01, 1.02)
**May-Sept only (N = 30,883)**^a^		
H_max_ (ref: <25) (n = 10,275)	1.00	1.00
25–29 (n = 8,567)	1.10 (1.03, 1.18)	1.10 (1.02, 1.18)
30–33 (n = 5,580)	1.12 (1.03,1.22)	1.12 (1.03, 1.22)
34 or greater (n = 6,461)	1.08 (0.99, 1.18)	1.08 (0.99, 1.18)
H_max_ (ref: <25) (n = 10,275)	1.00	1.00
25 or greater (n = 20,608)	1.10 (1.04, 1.18)	1.10 (1.03, 1.17)
H_max_	1.01 (1.00, 1.01)	1.01 (1.00, 1.01)
**Cherry harvest duties, Jun-Jul (N = 2,180)**^a^		
H_max_ (ref: <25) (n = 491)	1.00	1.00
25–29 (n = 652)	1.47 (1.09, 2.00)	1.43 (1.05, 1.94)
30–33 (n = 440)	1.77 (1.28, 2.46)	1.67 (1.20, 2.34)
34 or greater (n = 597)	1.68 (1.21, 2.33)	1.57 (1.11, 2.21)
H_max_ (ref: <25) (n = 491)	1.00	1.00
25 or greater (n = 1,689)	1.61 (1.23, 2.12)	1.53 (1.15, 2.02)
H_max_	1.03 (1.02, 1.05)	1.03 (1.01, 1.05)
**Apple harvest duties, Aug-Oct (N = 4,033)**^a^		
H_max_ (ref: <25) (n = 2,717)	1.00	1.00
25–29 (n = 789)	1.07 (0.87, 1.33)	1.07 (0.86, 1.32)
30–33 (n = 332)	1.05 (0.78, 1.41)	1.04 (0.77, 1.41)
34 or greater (n = 195)	0.77 (0.49, 1.22)	0.76 (0.48, 1.22)
H_max_ (ref: <25) (n = 2,717)	1.00	1.00
25 or greater (n = 1,316)	1.05 (0.87, 1.28)	1.05 (0.86, 1.28)
H_max_	1.02 (1.01, 1.03)	1.02 (1.01, 1.04)

H_max_ Maximum daily Humidex

^a^ Numbers (Ns and ns) refer to injury days and referent days

^b^ Adjusted for self-reported duration of employment at job of injury

## Discussion

In this case-crossover study of the relationship between heat exposure and traumatic injuries in outdoor agricultural workers, an increased risk of traumatic injuries was observed with increasing heat exposure to a maximum daily Humidex of approximately 33, after taking into account duration of employment. This finding is consistent with previous studies, conducted in different geographical areas and using different methods, that have reported an association between ambient temperature and injuries [17,18]. The magnitude of this risk per unit increase in exposure is roughly comparable in order of magnitude to the risk of injuries in the agricultural sector reported in previous studies [17,18], although direct comparisons are difficult because of differences in study design and primary heat exposure metrics. In addition, there was a particularly high risk of traumatic injuries in warm conditions during cherry harvest duties in the June and July time period, compared to all duties over the entire study period.

In the main analysis, a relative decrease in injury risk above a maximum daily Humidex of approximately 33 was observed. In Yakima, Washington, which is located in south central Washington, the 99^th^ percentile of daily high Humidex between 1980 and 2006 was 36 [34]. Xiang et al found an increased risk in daily injury claims up to a temperature of approximately 38°C, which is near the 95^th^ percentile of local maximum temperatures in Adelaide, Australia [18]. Adam-Poupart et al did not observe this “reverse U-shaped” relationship in Quebec, Canada [17]. It has been hypothesized that the observed differences in relationships may be related to different work practices and policies [17,18]. In some workplaces, work may cease when conditions become extremely hot.

In this study, injuries that occurred after 12:30 pm were less common on hotter days, possibly because some workers left work earlier or were dismissed earlier by employers on very hot days. Although injury times of day were accessible, information about work shift times was not accessible, and the exposure data source used in the study only contained maximum daily Humidex and air temperature values. In a sensitivity analysis excluding injuries that occurred after 12:30 pm, a trend of increasing odds ratios of injury with increasing exposure categories, including above a maximum daily Humidex of 33, was observed. The results of this sensitivity analysis lend support to the hypothesis that protective work practices (e.g. not working during the hottest parts of the afternoon) may reduce the risk of heat-related injuries. However, future studies that include individual-level information about work practices, hours, and heat exposure around the time of injury, for example from personal heat exposure sensors [35], could better address this hypothesis.

In addition to warm ambient conditions, internal heat generated by physical work, such as climbing ladders with bags of fruit, contributes to fatigue and heat stress [2]. A combination of heat exposure, dehydration, and fatigue can result in decreased postural stability and concentration [11,13,14]. Harvest workers are typically paid by the amount harvested (“piece rate”), except when crops are delicate. Piece rate pay, which incentivizes workers to work harder and faster, has been described to be associated with increased risks of heat-related illness [36] and injuries [37].

Cherry harvest duties that occurred during June and July were associated with a particularly high risk of traumatic injuries in warm conditions. The first intensive tree fruit harvest of the season in Washington is cherry harvest, which typically begins in June [21]. In this analysis, cherry harvest injuries were largely falls, had a higher prevalence of involving multiple body parts and missed work, and involved workers with relatively shorter durations of employment with the employer of injury than for all injuries. The threat of rainfall can create an additional demand for rapid cherry harvest and faster-paced work, as rain can cause cherries to absorb water and burst [21]. However, weather conditions that precede rain may be cooler, and this phenomenon would therefore be expected to bias results toward the null. In a post-hoc analysis, odds ratios of traumatic injury for peach and pear harvest duties that occurred during August and September were also high, but the number of injuries was small, and confidence intervals were wide (see S2 Table).

Associations between heat exposure and injuries for apple harvest occurring during the cooler August to October time period were less pronounced than for cherry harvest occurring during June and July. Apple harvest occurs later in the summer and in the fall, and workers may be better acclimatized by this time. In addition to manual harvest of apples using ladders, orchards are beginning to use mechanized harvest assist platforms, potentially requiring less physical exertion and resulting in less internal heat generation, and platform harvest may occur during the night [38]. The exact method of harvest was not available for this analysis.

Odds ratios of traumatic injury appeared generally lower after excluding injuries with more than seven days of time-loss and higher after excluding injuries with more than one day between the injury date and the first visit to a healthcare provider. More days of time-loss and more immediate healthcare access could be associated with more severe injuries and less rigorous employer health and safety procedures around the time of injury, which, if also associated with heat exposure, could confound the association between heat exposure and injuries. In secondary analyses, injuries with less than one day, compared to more than one day, between the injury date and the first visit to the healthcare provider were more likely to be characterized as injuries to the bones, nerves, and spinal cord (12% versus 8%) and multiple traumatic injuries (12% versus 9%) and less likely to be characterized as surface wounds and bruises (27% versus 38%) and soft tissue injuries (21% versus 34%). Employer health and safety procedures are not expected to vary widely over the course of the month-long referent window, and time invariant confounding is controlled for in the design of a case-crossover study. It is possible that the association between heat exposure and injury risk is generally lower for workers who work for employers with better health and safety programs. Xiang et al found an increased injury risk in hot conditions for injury claims associated with smaller employers [18]. Information about the safety practices of claimants’ employers was not available.

The odds ratio of injury for Humidex exposures of 30–33 was lower than in the primary analysis after excluding injuries with injury locations based only on the first healthcare provider’s address. The first healthcare provider’s address was used to impute injury location when there was missing data in the injury location field on the report of accident form and when the first healthcare provider was not located in the same county as the business location (see S1 Supplemental Material, p. 3). Relying on the first healthcare provider address as the injury location for these claims may have resulted in exposure misclassification. Workers who filed these claims may have been more likely to work in remote locations and have difficulty filling out the worker portion of the report of accident claim form due to language or other barriers. Future studies should investigate the risk of heat-related injury in these vulnerable sub-populations in more detail.

Interestingly, the prevalence of heat-related illness in the study population was very low. Heat-related illnesses are usually self-limited and may not result in treatment by a healthcare provider. Worker and healthcare provider recognition of heat-related illness is likely poor [8]. Even when recognized, the healthcare provider and worker may not report the illness to the workers’ compensation system. Workers with heat-related injuries likely present with a traumatic injury and heat-induced risk factors for injury such as dehydration, decreased vigilance, or heat-related illness. We hypothesize that in this case, the traumatic injury is the focus of the medical evaluation and treatment, with under-recognition of heat-induced risk factors for injury. It is also possible that heat-related injuries occur as a result of psychomotor decrements associated with mild dehydration [11] or other physiological phenomena before frank heat exhaustion and heat stroke develop. Studies that aim to elucidate the mechanism of the effect of heat exposure on injuries are needed.

### Strengths and limitations

This study is the first published study that we are aware of that focuses on heat exposure and traumatic injury risk in outdoor agricultural workers using modeled exposure data. The study has several important limitations that have not already been mentioned. First, detailed individual information about physical activity, an important contribution to fatigue, injury risk, and heat-related illness [2,13], was not available. It was not possible to extract information about all job duties associated with injury claims from the corresponding free text field on the report of accident claim form. This study focused on physically intense harvest duties. Second, the primary exposure, maximum daily Humidex, was computed from maximum daily temperature and mean daily humidity. Other humidity data were not available in the meteorological data source used in this study. However, Central/Eastern Washington is relatively dry, and sensitivity analyses using dry air temperature resulted in similar inferences. Third, this analysis focused only on new Washington State Fund agriculture traumatic injury claims in a State where a workplace heat standard was implemented, toward the end of the study period, in 2009 [31]. Results may not be generalizable to other Washington workers or to workers in other geographical regions. Future studies should assess whether heat policies and regulations are associated with reductions in heat-related injury rates.

## Conclusions

Outdoor agricultural workers performing physical work in warm ambient conditions are at increased risk of traumatic injuries. Further research is needed to elucidate the mechanism of this increased risk and to disentangle any contributions from earlier, warmer parts of the season and other factors associated with work duties. Additional analyses should be performed in other populations exposed to ambient heat to identify specific activities associated with the highest risks. Populations performing such activities may benefit from combined injury and heat-related illness prevention efforts with the goal of reducing heat-related injury and illness rates. The potential benefits of heat prevention interventions, including policies, should take into account reductions in morbidity, mortality, and costs associated with heat-related injuries in addition to other heat-related outcomes.

## Supporting Information

S1 Fig Traumatic injury claim identification. American National Standards Institute (ANSI); Occupational Injury and Illness Classification System (OIICS).
(TIF)

S2 Fig New Central/Eastern Washington adult traumatic outdoor agriculture injury cases. North American Industrial Classification System (NAICS); Standard Industrial Classification (SIC); Standard Occupational Classification (SOC) codes.
(TIF)

S3 Fig Locations of Central/Eastern Washington injuries by exposure level. (TIF)

S1 Minimal Dataset Minimal Dataset. (ZIP)

S1 Supplemental Material Additional methodological details. (DOCX)

S1 Table Frequencies of traumatic injuries during peak harvest months by harvest duty types. (DOCX)

S2 Table Odds ratios (ORs) and 95% confidence intervals (CIs) of workers’ compensation traumatic injuries for peach and pear harvest duties. (DOCX)
