# Supplementary figures and images for: A Case-Crossover Study of Heat Exposure and Injury Risk in Outdoor Agricultural Workers
Source: PLoS One. 2016 Oct 7;11(10):e0164498. doi: 10.1371/journal.pone.0164498 (PMC5055365; doi:10.1371/journal.pone.0164498)

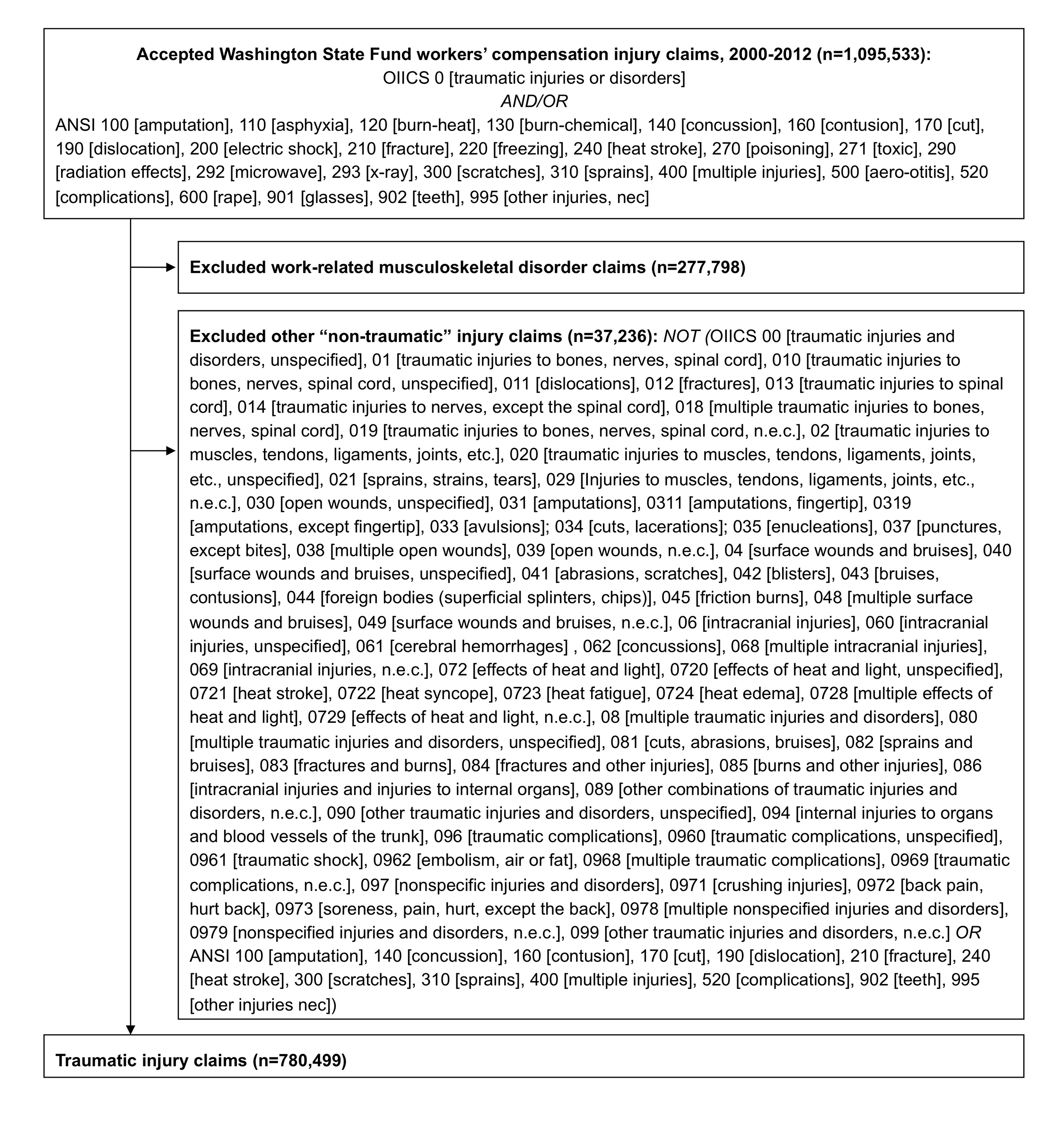

Supplement: S1 Fig — American National Standards Institute (ANSI); Occupational Injury and Illness Classification System (OIICS). (TIF) [file pone.0164498.s001.tif]

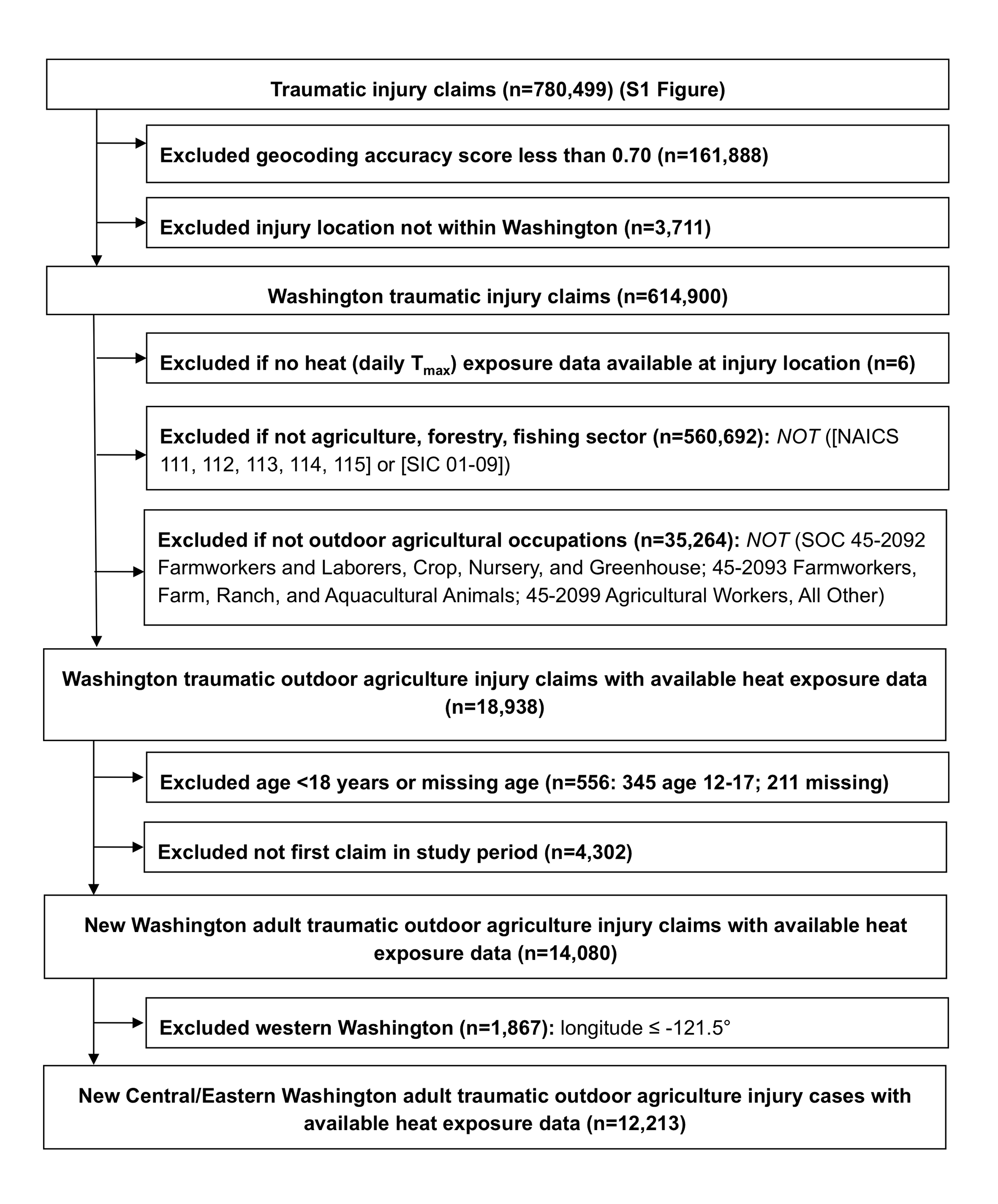

Supplement: S2 Fig — North American Industrial Classification System (NAICS); Standard Industrial Classification (SIC); Standard Occupational Classification (SOC) codes. (TIF) [file pone.0164498.s002.tif]

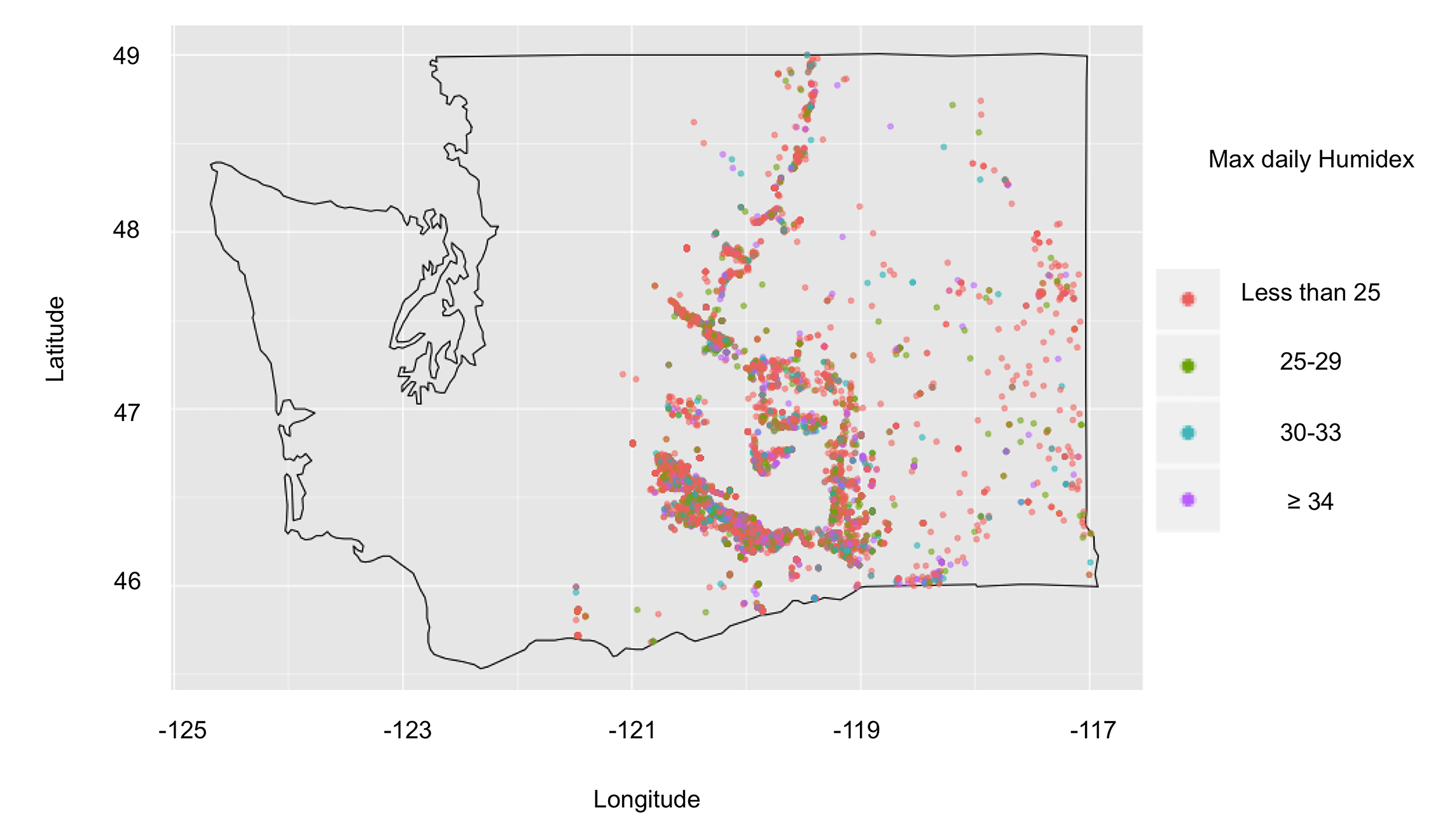

Supplement: S3 Fig — (TIF) [file pone.0164498.s003.tif]
