# Supplementary material for: A Case-Crossover Study of Heat Exposure and Injury Risk in Outdoor Agricultural Workers
Source: PLoS One. 2016 Oct 7;11(10):e0164498. doi: 10.1371/journal.pone.0164498 (PMC5055365; doi:10.1371/journal.pone.0164498)
Supplement: S1 Supplemental Material — (DOCX) [file pone.0164498.s005.docx]

**Additional Methodological Details**

**Workers’ compensation claims data**

Unless they are able to self-insure, employers in Washington State (with several exceptions, including the self-employed, federal government, those covered under other workers’ compensation systems, and household employers with one employee) are required to obtain workers’ compensation insurance through the Washington State Department of Labor and Industries (L&I) industrial insurance system [1]. L&I’s State Fund covers approximately two thirds of Washington State workers. This study was restricted to State Fund claims because limited information is available for self-insured claims that do not result in payment for time-loss (lost work time due to work-related injury or illness after a three day waiting period). The L&I claims management database consists of two main data processing systems: the Medical Information and Payment System, which receives all billing information generated by provider bills, and the L&I Industrial Insurance System, which contains data necessary for the administration of State Fund claims.

Data from injury workers’ compensation claims were extracted from L&I databases on August 20, 2015. Extracted information included date of injury, time of injury, sex, date of birth (to compute age at date of injury), height and weight (self-reported at claim opening and used to compute body mass index [BMI] [2]), addresses of the accident location and employer’s business location indicated on the claim form, the address and date of the first healthcare provider payee, six digit North American Industrial Classification System (NAICS) codes [3], three digit Standard Industrial Classification (SIC) System codes [4], six digit Standard Occupational Classification (SOC) codes [5], American National Standards Institute (ANSI) nature codes [6] and Occupational Injury and Illness Classification System (OIICS) nature, event/exposure, body part, and source codes [7], claim status (compensable time-loss versus medical treatment only claim codes), and time-loss days.

The number of days a worker reported working for the employer of injury at the time of injury was extracted from State Fund workers’ compensation claims databases. Washington Employment Security Department (ESD) data were also queried to determine whether claimants were reported to be working for the employer of injury (identified by Unified Business Identifier) during the quarter of injury and the quarter before the injury. ESD data are employer reports of wages and hours worked for each employee for federal and state unemployment insurance purposes. Apple and cherry harvest work duties were specifically identified using the *grepl* function in the base package in R 3.2.3 (R Foundation, Vienna, Austria) [8] (regular expressions used to isolate apple and cherry harvest duties were: "PICK.*APP|APP.*PICK|HARV.*APP|APP.*HARV" and "PICK.*CHER|CHER.*PICK|HARV.*CHER|CHER.*HARV", respectively).

**Traumatic injury claim identification**

Outdoor agriculture traumatic injury workers’ compensation claims were identified using the following procedure. A total of 1,095,533 injury claims, with injury dates between January 1, 2000 and December 31, 2012 that occurred either at the worksite or on the employer’s premises, were initially identified using ANSI and OIICS nature codes (S1 Figure). Before July 1, 2005, L&I used ANSI Z16.2 codes to classify injuries and illnesses based on the injury or illness narrative description on the workers’ compensation claim form that was submitted by the worker and physician [6]. For claims filed after July 1, 2005, L&I transitioned to coding injuries and illnesses using OIICS v1.01 codes [7]. During a transitional year from July 2005 to June 2006, claims were dual coded using ANSI and OIICS, and a crosswalk between ANSI and OIICS was optimized, allowing for compatibility and consistency between coding systems. Both ANSI and OIICS codes were available for all claims during the study period. Claims characterized using established case definitions [9] as work-related musculoskeletal disorders (WMSDs), or non-traumatic soft tissue disorders caused or aggravated by work activities, were excluded (n=277,798). Other non-traumatic injury claims (n=37,236) were excluded to yield 780,499 traumatic injury claims.

**Injury location assignment**

To assign a single location per claim as the injury location to which exposures were assigned, the following *a priori* scheme was used. If latitude and longitude were available for the accident location, the accident location was assigned to be the injury location (n=599,010; 77% of traumatic injury claims). If latitude and longitude were not available for the accident location, but the business location and the location of the first healthcare provider were in the same county and latitude and longitude were available for the business location, the business location was assumed to be the injury location (n=90,075, 12% of traumatic injury claims). Otherwise, the first provider location was imputed as the injury location, if latitude and longitude were available for the first healthcare provider’s location (n=89,675, 11% traumatic injury claims).

**Adult outdoor agriculture traumatic injury case definition**

Adult outdoor agriculture traumatic injury cases were defined using the scheme shown in S2 Figure. The geocoder used produces an accuracy score from 0 to 1 (with 1 indicating a perfect match) based upon Levenshtein distance for textual components, and parity for numerical components, of parsed address strings (as detailed in the file “database.rb” in the repository [10]). Claims with geocoding accuracy scores less than 0.70 for the location assigned to be the injury location were excluded (n=161,888, or approximately 21% of traumatic injury claims). Of the claims with an accuracy score of 0.70 or greater for the location assigned to be the injury location, latitude and longitude were derived from full address data for 65% of claims, from street level data for 7% of claims, at the city level for 13% of claims, and at the zip code level for 15% of claims.

Claims with injury locations outside of Washington, as defined using Federal Information Processing Standards (FIPS) county codes, were excluded (n=3,711). Of the remaining 614,900 Washington traumatic injury claims, six claims without daily heat exposure data available at the injury location were excluded. As this analysis focused on outdoor agricultural workers, claims that were not characterized as within the agriculture/forestry/fishing sector were excluded (n=560,692), and claims that were not characterized as outdoor agricultural occupations [11], were also excluded (n=35,264).

Of the Washington traumatic outdoor agriculture injury claims with available heat exposure data (n=18,938), 345 characterized by claimant age less than 18 or with missing age values (n=211) were excluded from our study. Less than 2% of outdoor agricultural workers’ injury claims occurred in workers younger than 18 years. Washington employment rules and the potential for workers’ compensation claim filing are different in workers under 18 years compared to adults. These differences have implications for the assessment of injury risk in this younger population using our existing data sources. For example, according to child labor laws for agricultural employment in Washington State, minors (workers under age 18) are prohibited from performing certain hazardous work tasks and from working during certain hours of the night [12,13]. Twelve and 13 year-olds are allowed to work only during non-school weeks in hand-harvesting berries, bulbs, cucumbers and spinach. Fourteen through 17 year-olds are allowed to work limited hours during school weeks. As the analysis focused on new injury claims during the study period, repeat injury claims during the study period were excluded (n=4,302). We also excluded claims in Western Washington (n=1,867) in the primary analysis.

**References**

1. Revised Code of Washington. Employments and Occupations Covered [Internet]. Olympia, WA: Washington State Legislature; 2005 [cited 2016 Jul 1]. Available from: http://apps.leg.wa.gov/rcw/default.aspx?cite=51.12.

2. CDC. How is BMI calculated and interpreted? About BMI for adults. [Internet]. Atlanta, GA: Centers for Disease Control and Prevention; 2014 [cited 2016 Jul 1]. Available from: http://www.cdc.gov/healthyweight/assessing/bmi/adult_bmi/index.html#Interpreted.

3. US Census Bureau. North American Industry Classification System [Internet]. Suitland, Maryland: US Department of Commerce; 2012 [cited 2016 Jul 1]. Available from: http://www.census.gov/eos/www/naics/index.html.

4. US Department of Labor. Standard Industrial Code Manual [Internet]. Washington, DC: Occupational Safety & Health Administration; 2012 [cited 2016 Jul 1]. Available from: http://www.osha.gov/pls/imis/sic_manual.html.

5. US Department of Labor. Standard Occupational Classification (2000) [Internet]. Washington, DC: US Bureau of Labor Statistics; 2010 [cited 2016 Jul 1]. Available from: http://www.bls.gov/soc/home.htm.

6. ANSI. Methods of recording basic facts relating to the nature and occurrence of work injuries. New York, NY: American National Standards Institute; 1969.

7. NIOSH. Occupational injury and illness classification system [Internet]. Atlanta, GA: Centers for Disease Control and Prevention; 2015 [cited 2016 Jul 1]. Available from: http://wwwn.cdc.gov/wisards/oiics/Trees/MultiTree.aspx?Year=2007.

8. R Development Core Team. R: A Language and Environment for Statistical Computing [Internet]. Vienna, Austria: R Foundation for Statistical Computing; 2011 [cited 2016 Jul 1]. Available from: http://www.r-project.org.

9. Anderson N, Adams D, Bonauto D, Howard N, Silverstein B. Work-related musculoskeletal disorders of the back, upper extremity, and knee in Washington State, 2002-2010 [Internet]. Olympia, WA: Washington Department of Labor and Industries Safety and Health Assessment and Research for Prevention Program; 2015 [cited 2016 Jul 1]. Available from: http://www.lni.wa.gov/safety/research/files/wmsd_techreport2015.pdf.

10. Erle S. Geocoder::US [Internet]. San Francisco, CA: GitHub; 2016 [cited 2016 Jul 1]. Available from: https://github.com/geocommons/geocoder.

11. Crider KG, Maples EH, Gohlke JM. Incorporating occupational risk in heat stress vulnerability mapping. J Environ Health. 2014;77(1):16–22.

12. US Department of Labor. State child labor laws applicable to agricultural employment [Internet]. Washington, DC: Wage and Hour Division; 2016 [cited 2016 Aug 10]. Available from: <https://www.dol.gov/whd/state/agriemp2.htm#Washington>.

13. Washington Department of Labor and Industries. Young workers in agriculture [Internet]. Olympia, WA: Washington Department of Labor and Industries; 2010 [cited 2016 Aug 19]. Available from: http://www.lni.wa.gov/IPUB/700-096-909.pdf.
