# Supplementary material for: A Case-Crossover Study of Heat Exposure and Injury Risk in Outdoor Agricultural Workers
Source: PLoS One. 2016 Oct 7;11(10):e0164498. doi: 10.1371/journal.pone.0164498 (PMC5055365; doi:10.1371/journal.pone.0164498)
Supplement: S1 Table — (DOCX) [file pone.0164498.s006.docx]

**S1 Table. Frequencies of traumatic injuries during peak harvest months by harvest duty types**

| **Month** | **Apple harvest duties^a^** | **Cherry harvest duties^b^** | **Peach and pear harvest duties^c^** |
| --- | --- | --- | --- |
| June |  | 305 |  |
| July |  | 241 |  |
| August | 97 |  | 57 |
| September | 439 |  | 71 |
| October | 445 |  |  |

^a^Peak harvest months August-October (94% apple harvest injuries)

^b^Peak harvest months June-July (96% cherry harvest injuries)

^c^Peak harvest months August-September (93% peach and pear harvest injuries)
