# Supplementary material for: A Case-Crossover Study of Heat Exposure and Injury Risk in Outdoor Agricultural Workers
Source: PLoS One. 2016 Oct 7;11(10):e0164498. doi: 10.1371/journal.pone.0164498 (PMC5055365; doi:10.1371/journal.pone.0164498)
Supplement: S2 Table — (DOCX) [file pone.0164498.s007.docx]

**S2 Table. Odds ratios (ORs) and 95% confidence intervals (CIs) of workers’ compensation traumatic injuries for peach and pear harvest duties**

|  | **Unadjusted OR (95% CI)** | **Adjusted OR (95% CI)**^b^ |
| --- | --- | --- |
| **Peach and pear harvest duties, Aug-Sept (N=499)**^a^ |  |  |
| H_max_ (ref: <25) (n=197) | 1.00 | 1.00 |
| 25-29 (n=144) | 1.71 (1.03, 2.82) | 1.84 (1.10, 3.06) |
| 30-33 (n=77) | 1.31 (0.68, 2.54) | 1.47 (0.74, 2.90) |
| 34 or greater (n=81) | 1.29 (0.63, 2.64) | 1.56 (0.73, 3.35) |
| H_max_ (ref: <25) (n=197) | 1.00 | 1.00 |
| 25 or greater (n=302) | 1.55 (0.97, 2.48) | 1.72 (1.06, 2.80) |
| H_max_ | 1.02 (0.98, 1.06) | 1.03 (0.99, 1.08) |

H_max_ Maximum daily Humidex

^a^Numbers (Ns and ns) refer to injury days and referent days

^b^Adjusted for self-reported duration of employment at job of injury
